# Elephantiasis mimicry in recurrent lower limb skin infections in a diabetic patient: a case report

**DOI:** 10.1186/s13256-023-04260-x

**Published:** 2023-12-16

**Authors:** Puneet Bramania, Emmaeli Moshi, Andrew Foi, Grace Shayo

**Affiliations:** 1 Department of Internal Medicine, Muhimbili University of Health and Allied Sciences, Dar es Salaam, Tanzania; 2 Department of Pathology, Muhimbili National Hospital, Dar es Salaam, Tanzania; 3 Department of Internal Medicine, Dermatology Unit, Muhimbili National Hospital, Dar es Salaam, Tanzania

**Keywords:** Elephantiasis, Skin infections, Lymphedema, Diabetes mellitus, Case report

## Abstract

**Background:**

Chronic edema as a complication of systemic diseases or infections can mimic filarial lymphedema (also known as elephantiasis) and considered so. We describe a case of chronic lymphedema that mimicked elephantiasis in a diabetic man.

**Case presentation:**

The patient was a 70-year-old black man, bed-bound at the time of admission following a diagnosis of stroke and hypertension in the previous 5 years. He had been diabetic for 20 years with poorly controlled diabetes mellitus. He suffered recurrent bilateral lower limb skin infections for 5 years prior to admission that culminated into progressive lowerlimb edema. The infections eventually complicated into skin edema, hardening, fissuring, and hyperkeratotic plaques. The physical examination revealed Tinea pedis and bilateral non-pitting edema of lowerlimbs to the level of the knees. Investigations confirmed non-filarial lymphedema-related skin changes. The absence of the classic pebbly/cobblestone skin changes ruled out elephantiasis nostra verrucosa (ENV), with a possibility of it being in the early stages of evolution. The patient’s skin fissuring and infections were successfully treated with antibiotics and antifungals while compression stockings helped to relieve the edema.

**Conclusions:**

Chronic lymphedema can complicate repeated non-filarial infections of lower limbs. Its fissures are a risk factor for cellulitis, prompting early identification and management of both infections and lymphedema to halt their vicious cycle, especially in at risk populations like diabetics.

## Introduction

Lymphedema is the swelling of part of the body usually the arms or legs as a result of protein-rich interstitial volume overload secondary to impaired lymphatic drainage [1]. Elephantiasis is the abnormal enlargement and disfigurement of a part of the body secondary to chronic lymphedema [1, 2]. Lymphatic filariasis is a common cause of elephantiasis especially in tropical countries like Tanzania [1]. Elephantiasis is accompanied by chronic inflammation, recurrent skin infections, and subsequent fibrosis in the skin and subcutaneous tissues [1–3]. These processes result in thickening, hardening, papillomatosis, verrucous, and cobblestone-like appearance of the skin [4–6]. Filarial lower limb lymphedema is usually unilateral, unlike non-filarial bilateral lymphedema which can occur as a sequela of long-standing edema from systemic conditions like heart failure or renal failure. Venous insufficiency is also known to cause lymphedema. Delayed diagnosis and treatment can lead to progressive skin fibrosis, fissuring, recurrent cellulitis, papillomatosis, and verruca- like skin changes [1, 2].

The term elephantiasis nostras verrucosa (ENV) describes chronic lymphedema with its accompanied verrucous skin changes [1, 4, 5]. It has been reported in patients with edema secondary to chronic venous insufficiency, heart failure, trauma, radiotherapy, pretibial myxedema or congenital lymphedema [2, 5–8], thus largely presenting as a bilateral disease. We report a case of an elderly man who presented with bilateral chronic edema that mimicked elephantiasis in the context of uncontrolled diabetes mellitus. This case report will remind clinicians and care takers to promptly treat lower limb infections in diabetics to avoid similar complications.

## Case presentation

### Patient Information

In 2021 we admitted a bed-bound 70 year- old black man of African origin with type 2 Diabetes mellitus (DM) for more than 20 years and hypertension for 5 years. The hypertension was diagnosed when he presented with a stroke that resulted into left hemiplegia. His recent medications were Metformin 500 mg daily and Nifedipine 20 mg daily. He had poor adherence to therapy, and infrequent monitoring of blood glucose levels.

His main complaints were a prolonged history of recurrent skin problems involving both legs since the time he became bed-ridden after the stroke. Chronologically, these occurred initially as poorly healing pressure ulcers on already numb heels of both feet. He had then gotten at least five events of cellulitis of the legs and feet that had been treated twice with intravenous antibiotics (as an inpatient), and other times with oral antibiotics. During this course, he had also been repeatedly treated for fungal infections of the toes.

The major primary concern for the index admission was a progressive swelling of both lower limbs and disfigurement of its skin. Both legs were swollen below the knees, which he had noticed for the past 3 years. Limb rising did not significantly relieve the swelling that had become persistent. About 2 years before the current admission, he had been admitted for poorly healing wounds at the calves that were complicated by infection and myiasis. This was subsequently treated with multiple sloughectomies and antibiotics.

About 6 months prior to the current admission he had noticed progressive skin changes on both shins and calves that were hard and dry, with a rough surface. This was accompanied by recurrent itching, pain, and discomfort. There were no similar skin changes or swelling in the genitalia or elsewhere in the body. He had no history of fevers or lumps in his inguinal area. His course was complicated by a painful blister on the sole of the left foot which ruptured spontaneously releasing yellowish fluid, followed by skin peeling. The review of other systems was unremarkable for any significant complaints. Specifically he did not present with symptoms of hypothyroidism or hyperthyroidism. He presented with symptoms of prostatism but had normal urine color and amount.

He had no history of trauma or exposure to radiations to the lower limbs. He denied a history of walking bare feet at the workplace. He was a retired construction site supervisor residing in the largest metropolitan city in Tanzania, Dar es Salaam. He had been living with his brother’s family who took care of him. His wife and children deserted him following his disability from the stroke. He had quitted alcohol use and smoking 5 years ago. Many years ago he had a penile painless ulcer that was diagnosed and treated as syphilis. He had no history of any major surgeries or blood transfusions. His family history was consistent with DM and hypertension.

### Clinical findings

**Fig. 1:**
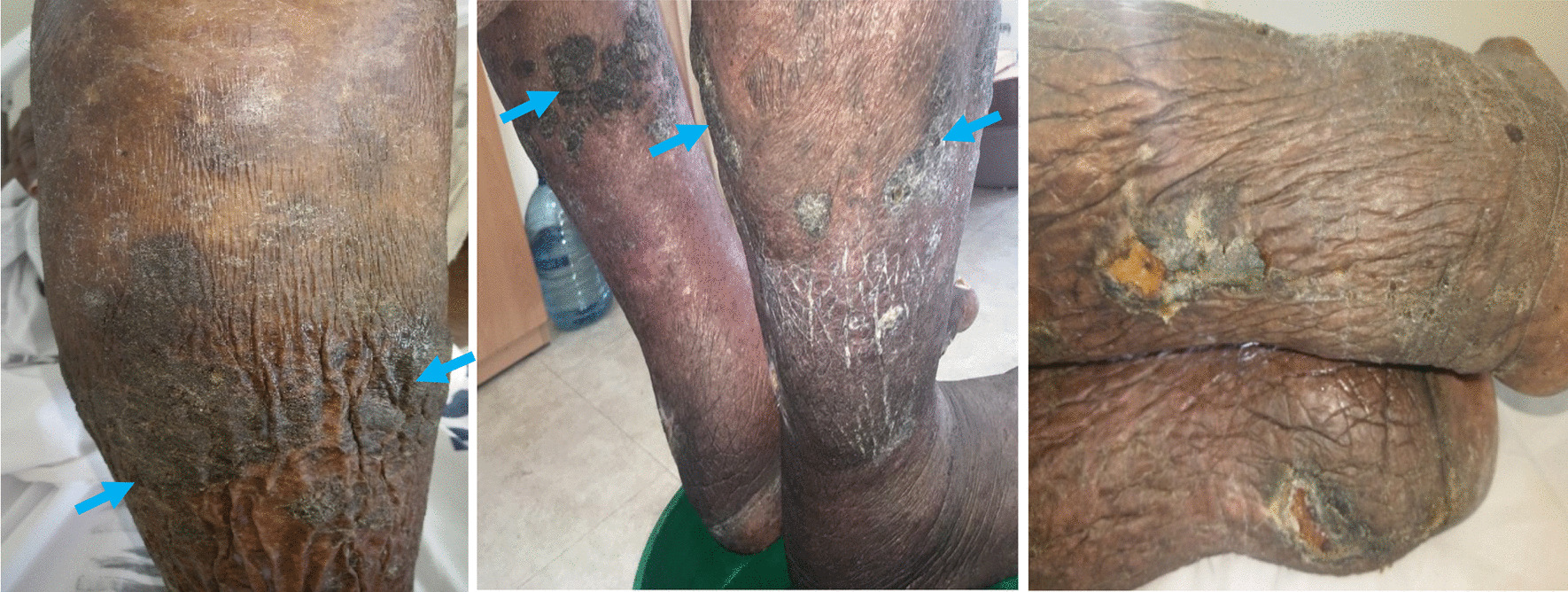
Hyperkeratotic plaques (arrows), irregular lichenified skin folds with fissures and crusted ulcers on both legs

**Fig. 2:**
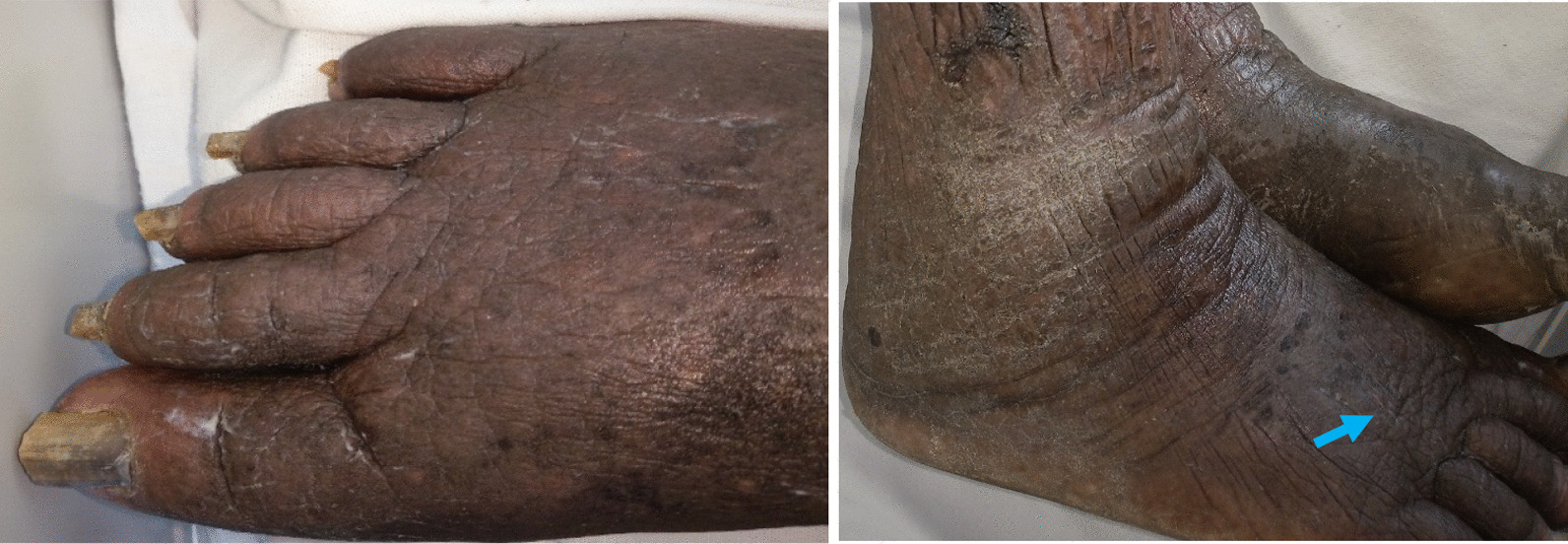
Edematous, lichenified feet with dystrophic nails, and early cobblestone-like changes (arrow)

General examination revealed an elderly, fully conscious, unkempt man. He did not appear wasted. Both lower limbs revealed non-tender, non-pitting edema up to the knee level. Few mobile, non-tender, approximately 1 to 2 cm inguinal lymph nodes were palpable on both sides. There was no scrotal or penile swelling or ulcer. Examination of the integumentary system of the lower limbs was as follows:

(i) Legs: Hard, dry, rough, and lichenified skin with some fissures on both legs below the knees. There were hyperkeratotic, hyper-pigmented, hard, dry, and rough-textured plaques with irregular borders on both anterior and posterior aspects of both legs. There were shallow ulcers with yellow base and crusted scarred margins on posterior surface of both legs (Fig. 1). The ulcers had no discharge or any signs of active inflammation on the surrounding skin. There were no visible distended veins on both legs, and distal pulses were feeble.

(ii) Feet: The skin on the dorsal surfaces of both feet was lichenified, hard, and non-pitting with a positive Kaposi-stemmer sign (could not pinch the skin of the second toe). Early cobblestone-like changes were noted on the right foot (Fig. 2). The plantar surfaces of both feet had thick skin with no ulcers or fissures. There was a loss of fine touch, temperature, and vibratory sensation. (iii) Toes: The toe nails of both feet were thick, dystrophic, yellow colored, but there was no onycholysis or subungual hyperkeratosis. The interdigital toe spaces had macerated whitish plaques with fissures.

Other systemic examination revealed grade 1 hypertension, tachycardia, spastic left hemiparesis, distal sensory neuropathy, and firm prostatomegaly grade 1.

### Diagnostic assessment

Blood investigations included: (i) Complete blood count that showed leukocytosis (total white cell count of 16.8 × 10^9^/l with absolute neutrophils of 13.6 × 10^9^/l (80.9%), microcytic hypochromic anemia (hemoglobin of 10.8 g/dl, MCV = 67.1 fl, MCH = 22 pg, MCHC = 32.8 g/dl), and thrombocytosis (platelet count of 558 × 10^12^/l), (ii) Inflammatory markers (C-reactive protein 134.7 mg/l, Ferritin 412.6 ng/ml) were raised, (iii) Fasting blood glucose was 10.1 mmol/l and glycated hemoglobin was 8.4%, (iv) Biochemistry tests showed normal renal, liver, and thyroid functions.

**Fig. 3:**
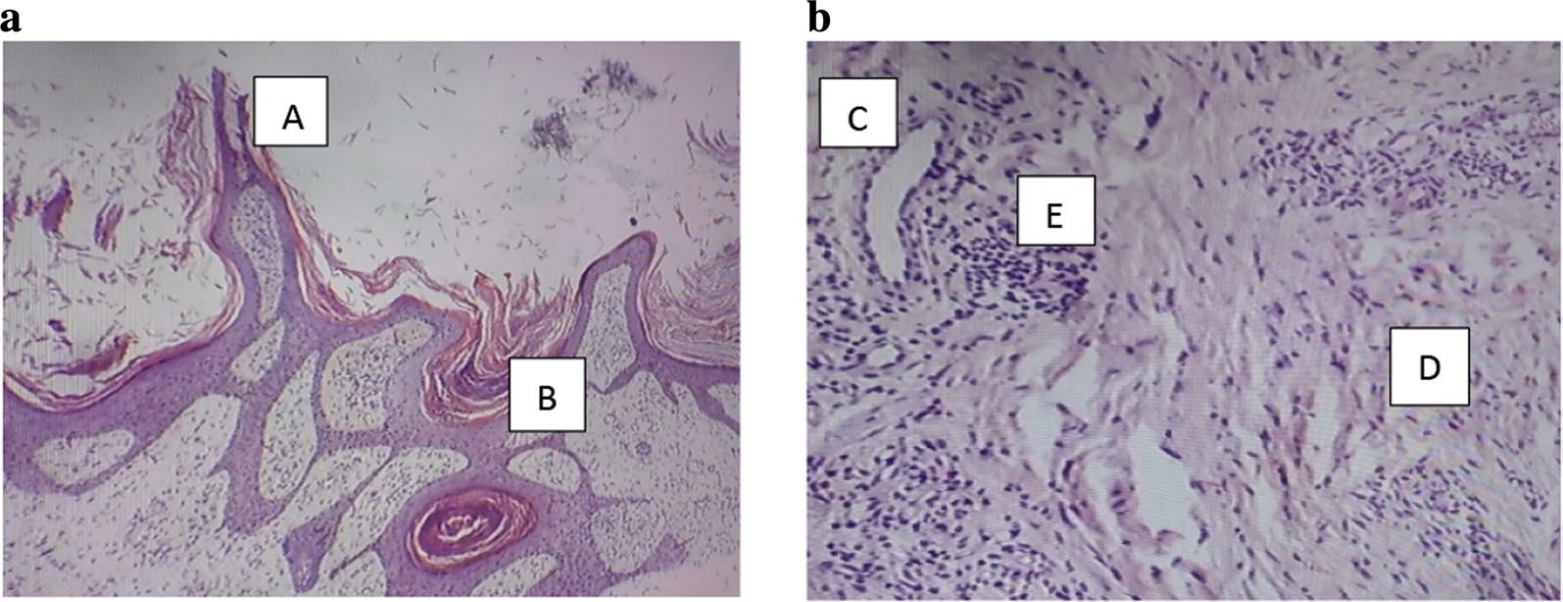
**a** Haematoxyline and Eosine stained view. **b** High magnification (×40) view of skin biopsy showing Acanthopapilloma [A], marked hyperkeratosis [B] with Dilated lymphatic channels [C], dermal fibrosis [D] and lymphocytic infiltrates [E]

Blood and swab (from ulcer base) cultures did not show any growth. Fungal hyphae were detected from potassium hydroxide (KOH) preparation of nail crumbling. A peripheral blood smear done at 12 mid-night did not show any microfilariae. Vascular Doppler sonography of both lower limbs revealed normal veins and some arterial atherosclerotic changes without significant luminal narrowing. A lymphoscintigraphy scan of lower limbs was not done due to inavailability at the hospital and failure to afford it at a peripheral center. A skin biopsy from the periphery of the hyperkeratotic plaque was assessed by a pathologist experienced in interpreting skin biopsies. It showed marked hyperkeratosis with some acanthopapilloma, multiple ectatic lymphatics, perivascular lymphoplasmacytic infiltrates, and patchy areas of fibrosis in the dermis (Fig. 3).

Cardiovascular assessment revealed a sinus tachycardia in an electrocardiogram while an echocardiogram showed mild concentric left ventricular hypertrophy with an ejection fraction of 63%, grade 1 diastolic dysfunction, and sclerotic aortic valve. An abdominal ultrasound revealed enlarged prostate with an intact capsule.

### Diagnosis, therapeutic interventions, and clinical outcome

With reference to the above workup, we concluded the final diagnosis as uncontrolled Type 2 Diabetes mellitus and hypertensive heart disease presenting with elephantiasis-like edema and dermatophytosis complex. The patient was given medical treatment and care by a multi-disciplinary team involving endocrinologist, dermatologists, physicians, physiotherapists, nurses, and social workers. Diabetes mellitus was treated with insulin therapy and metformin and fasting blood glucose normalized. Losartan-hydrochlorothiazide, oral paracetamol as an analgesic, pregabalin, and multi-vitamin supplements were also prescribed.

**Fig. 4:**
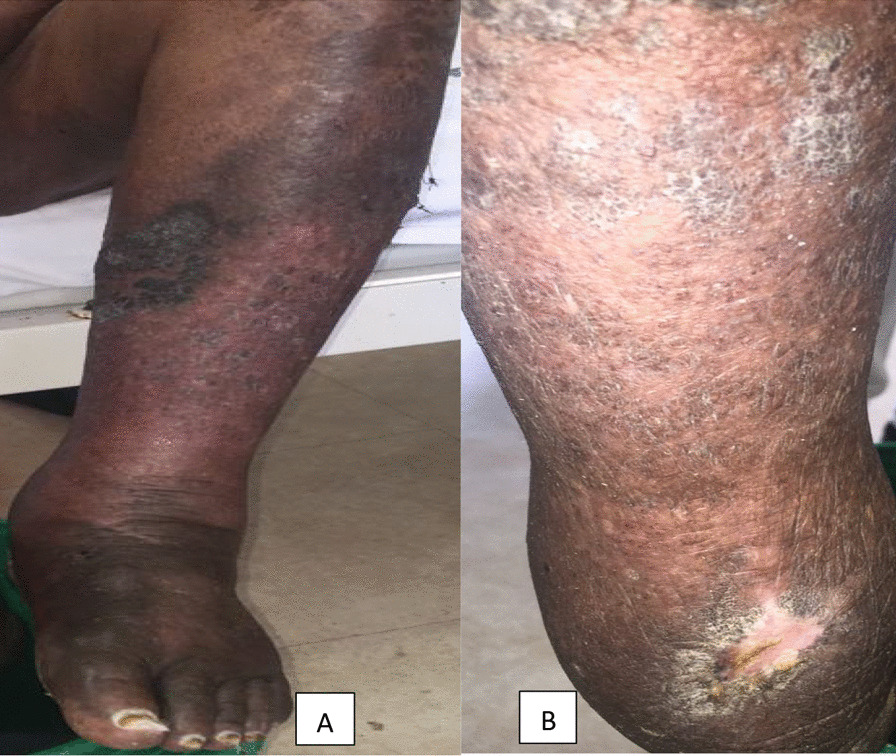
Improvement in skin lesions after treatment with compression bandaging, massaging, topical keratolytic, emollients, and antibiotics. **A** Anterior surface of the left leg showing patchy areas of hyper pigmented and lichenified skin. **B** Posterior surface of the left leg showing lichenified skin, a healed ulcer with scar and crust just above the left ankle

Foot and skin care was provided by nurses as instructed by the dermatologists and endocrinologists. The ulcers were cleaned with povidone and normal saline and the patient’s legs were dipped in diluted potassium permanganate solution for at least 30 min daily. Tight compression bandaging of both legs was done up to the lower half of the thighs. Topical mupirocin cream was applied to the ulcers and wounds, topical salicylic acid 15% (keratolytic) cream was applied on the hyperkeratotic plaques, and emollients massaged on the entire legs. The physiotherapist assisted in limb movements and ambulation. Intravenous piperacillin-tazobactam 3.375 g 6 hourly for 7 days followed by oral amoxicillin-clavulinic acid 625 mg twice daily for 5 days and, oral fluconazole 200 mg daily for 2 weeks were also prescribed. Interdigital toe webs were cleaned with povidone and normal saline followed by application of terbinafine cream for at least 2 weeks. The patient fared well with a good control of blood glucose, blood pressures, pain, and infection. The ulcers healed after a prolonged period of almost 4 weeks. The hyperkeratotic plaques and interdigital plaques vanished gradually, however some areas of hyperpigmentation, lichenification, scarring, and the irregular hard texture of the skin persisted (Fig. 4). The prognosis of the patient was initially fair given a well control of infections, diabetes and hypertension. The patient felt much better psychologically from the improvement of the disfigured skin on the legs, though his physical frailty did not fully improve. After about a month of inpatient care, the patient was discharged on monthly intramuscular benzathine penicillin 2.4 MIU to prevent early recurrence of cellulitis. It was unfortunate that the patient got lost to follow up immediately after discharge, making his prognosis poor.

## Discussion

We have described the case of a 70 year-old man with chronic edema that mimicked elephantiasis. This case was thoroughly investigated to search for the cause of edema and only infections in the midst of uncontrolled diabetes mellitus were linked to it. Failure to do lymphoscintigraphy hindered us to understand the extent of damage to the lymphatic system.

Although in some cases chronic edema might be the initiator of the cascade of infections, repeated bacterial infections from skin abrasions can lead to chronic lymphedema as a complication of acute dermatolymphangioadenitis (ADLA) [9] or chronic lymphangitis, the two (lymphedema and infections) creating a vicious cycle. Such a complication has been described in Elephantiasis nostras verrucosa (ENV), a consequence of chronic inflammation of the skin that occurs in chronic lymphedema. ENV was first described in 1934 by Aldo Castellani, an Italian microbiologist [4]. It is accompanied by verrucous and cobblestone-like skin changes that are a consequence of chronic inflammation of the skin that occurs in chronic lymphedema [4–6].

We have described an elderly male patient with uncontrolled DM who succumbed to diabetic foot complications including recurrent fungal infections that complicated into recurrent cellulitis and possibly lymphangitis. Similar cases have been reported in a patient with diabetic neuropathy [10] and in an elderly diabetic patient with recurrent erysipelas [11]. Recurrent skin infections can lead to chronic lymphangitis that culminates in lymphedema [1, 3]. Lymphedema in turn weakens the local immune defenses of the skin (both physically through skin fissuring and physiologic diminution of immune processes) predisposing to recurrent cellulitis thriving the vicious cycle [3]. Ulcerations, skin fissures, crusts, and interdigital maceration acts as an entry point and nidus for colonization of bacteria and fungi which then induce recurrent cellulitis and lymphangitis [1–3]. These were all evident in the patient we described. Lymph stasis from lymphangitis results in the accumulation of lymphocytes, proteins, fibroblast proliferation which ultimately causes chronic skin inflammatory changes [1–3].

In our case, this inflammatory process was indicated by raised C-reactive protein (CRP) and ferritin levels, and skin biopsy findings of lymphoplasmacytic infiltrates and dermal fibrosis. Our patient had features of stage 3 elephantiasis but did not present with the classic signs of ENV. We did not find the obvious cobblestone-like appearance of the skin (except in a small area on the dorsum of the foot), and the characteristic skin biopsy finding of pseudoepitheliomatous hyperplasia reported in cases of ENV [5, 6]. We propose that our patient was at an early stage of ENV that was likely in transition to the pebbly and cobblestone appearance. Though lymphoscintigraphy was not done in our case, we attempted an extensive search to exclude other causes of the lymphedema. Vascular Doppler sonography of lower limbs did not show any features of venous insufficiency. Heart and renal failure were excluded by the absence of relevant symptoms and normal renal function respectively. There was no mention of any limb surgery or surgical removal of lymph nodes, trauma, or radiation exposure. Podoconiosis was unlikely due to the lack of exposure to micro-particles from walking barefoot. The absence of distinguishing skin histopathology findings of other rare causes of ENV (for example, lipedema, lipodermatosclerosis, papular mucinosis, and cutis carcinoides) excludes these conditions [5, 8]. Pretibial myxedema was unlikely in the setting of a normal thyroid panel.

The treatment of this chronic edema is challenging, the skin may not completely revert to normal. Compression therapy with massage, multi-layer inelastic bandaging, and tight compressive stocking are the cornerstone in reducing the swelling [1, 12]. Besides, the underlying cause should be searched and treated accordingly [5, 12]. Adequate skin hygiene and treatment of local infections should be provided [1, 12]. Lymphedema is a risk factor for cellulitis. The vicious cycle of recurrent cellulitis and lymphedema as described above should be stopped [3]. Untreated and complicated tinea pedis in diabetic patients may predispose to additional bacterial colonization increasing the risk for cellulitis, and therefore needs to be timely and appropriately treated [3, 13]. The skin changes and infections were well treated in our case. Monthly benzathine penicillin injections have shown to reduce recurrence of cellulitis [13]. However, monthly benzathine penicillin and further regular assessment was not possible due to socio-economic limitations and loss to follow up.

## Conclusion

The chronic edema described in this case is a rare complication of repeated infections in the lower limbs of an elderly man with poorly controlled diabetes mellitus. It might have been ENV in evolution, as there was no classic cobblestone appearance of the skin seen in ENV. Skin infections and foot complications in diabetic patients need be promptly identified and treated to prevent lymphedema and its related skin complications.

## Data Availability

Information related to the case is available from the corresponding author upon a reasonable request.

## Abbreviations

CRP: C-reactive protein
DM: Diabetes mellitus
ENV: Elephantiasis nostra verrucosa
MCH: Mean cell volume
MCHC: Mean cell hemoglobin concentration
MCV: Mean cell volume
MIU: Million International Units
